# Supplementary material for: Identifying and Understanding the Non-Clinical Impacts of Delayed or Cancelled Surgery in Order to Inform Prioritisation Processes: A Scoping Review
Source: Int J Environ Res Public Health. 2022 May 3;19(9):5542. doi: 10.3390/ijerph19095542 (PMC9103788; doi:10.3390/ijerph19095542)
Supplement: Supplementary file 1 [file ijerph-19-05542-s001.zip › ijerph-1591736-supplementary.pdf]

Supplementary File S1. Twelve Papers rejected for inclusion.

Publication	Country	Population	Study Design	Key Findings	Reasons for Rejection
Gustafsson et al., 2010	Sweden	16 people > 65 years waiting for hip or knee replacement	Qualitative	The "meaning of care" with both formal and informal carers' roles discussed	Focus on the meaning of care rather than the experience of waiting
Robling et al., 2009	UK (Cardiff)	36 people waiting for arthroplasty (n=20) or specialist opinion	Qualitative	Four themes identified, none about patient's experiences of any harms	Focus on the healthcare system and the doctor's role
Al-Jabir et al., 2020a	UK (London)	Surgical practice during the Covid-19 pandemic	Narrative review	Focus on practical issues such as PPE, infection control, education, research.	An overview of guidelines with focus on the practicalities of service delivery
van der Horst et al., 2019	Amsterdam	12 people perioperative lumbar fusion	Qualitative	Focus on post-operative pain and coping	No details on non-clinical or social harms
Curtis et al., 2010	Australia	People waiting for elective surgery	Narrative review	Key elements needed for a prioritisation system are; equity, transparency, certainty, valid/reproducible.	No details on non-clinical or social harms
Testi et al., 2008	Italy	Surgical waiting lists	Modelling exercise	Urgency based on clinical conditions and is a mix of disease progression, pain/dysfunction, and disability. Priority for surgery takes into account the time on waiting list.	No details on non-clinical or social harms
Nuñez et al., 2006	Spain	People waiting for knee replacement	Randomised Controlled Trial	Improvements in pain, stiffness, function after a three-month exercise intervention. People also took their analgesia properly which confounds the effects.	No details on non-clinical or social harms
Wallis & Taylor, 2011	Australia	Pre- operative interventions for people waiting for hip/knee replacements	Systematic review and meta-analysis	Low to moderate evidence from small RCTs shows that pre op exercise can reduce pain and that pre-operative exercise and education may improve activity after hip replacement	No details on non-clinical or social harms
Al-Jabir et al., 2020b	UK (London)	people waiting for surgery during Covid-19 pandemic	Review of different specialities working practice	Focus on clinical (physical) prioritisation and changes to staff practice to minimise infections	No details on non-clinical or social harms
Oudhoff et al., 2007	Netherlands	People waiting for varicose veins removal, hernia repair and cholecystectomy. Pre- and post-operative questions, 27 hospitals.	Cross sectional questionnaire, pre and post op	Pain increased and mobility decreased whilst waiting. Length of wait was not associated with problems whilst waiting. Up to 41% of patents had minimal symptoms while waiting.	No details on non-clinical or social harms
Chan et al., 2012	Hong-Kong	11 studies on patients' pre-operative experiences.	Systematic review	Four themes identified: need for information, involving relatives, need for control, and healthcare professionalism	No details on non-clinical or social harms
Walker et al., 2016	Canada	590 women waiting for gynaecological surgery	Cross-sectional survey	High burden of pre-operative pain. 61% sought healthcare advice, 33% had moderate/severe pain	No details on non-clinical or social harms

Al-Jabir, A, Kerwan, A., Nicola, M., Alsafi, Z., Khan, M., Sohrabi, C., O'Neill, N., Iosifdis, C., Griffin, M., Mathew, G., & Agha, R. (2020). A commentary on "Impact of the Coronavirus (COVID-19) pandemic on surgical practice - Part 1". *International Journal of Surgery*, *79*(January), 168–179. https://doi.org/https://doi.org/10.1016/j.ijsu.2020.05.002

Al-Jabir, Ahmed, Kerwan, A., Nicola, M., Alsafi, Z., Khan, M., Sohrabi, C., O'Neill, N., Iosifidis, C., Griffin, M., Mathew, G., & Agha, R. (2020). Impact of the Coronavirus (COVID-19) pandemic on surgical practice - Part 2 (surgical prioritisation). *International Journal of Surgery*, *79*(May), 233–248. https://doi.org/10.1016/j.ijsu.2020.05.002

Chan, Z., Kan, C., Lee, P., Chan, I., & Lam, J. (2012). A systematic review of qualitative studies: Patients' experiences of preoperative communication. *Journal of Clinical Nursing*, *21*(5–6), 812–824. https://doi.org/10.1111/j.1365-2702.2011.03942.x

Curtis, A. J., Russell, C. O. H., Stoelwinder, J. U., & McNeil, J. J. (2010). Waiting lists and elective surgery: Ordering the queue. *Medical Journal of Australia*, *192*(4), 217–220. https://doi.org/10.5694/j.1326-5377.2010.tb03482.x

Gustafsson, B. Å., Heikkilä, K., Ekman, S. L., & Ponzer, S. (2010). In the hands of formal carers: Older patients' experiences of care across the perioperative period for joint replacement surgery. *International Journal of Orthopaedic and Trauma Nursing*, *14*(2), 96–108. https://doi.org/10.1016/j.ijotn.2010.01.002

Nuñez, M., Nuñez, E., Segur, J. M., Macule, F., Quinto, L., Hernandez, M. V., & Vilalta, C. (2006). The effect of an educational program to improve health-related quality of life in patients with osteoarthritis on waiting list for total knee replacement: A randomized study. *Osteoarthritis and Cartilage*, *14*(3), 279–285. https://doi.org/10.1016/j.joca.2005.10.002

Oudhoff, J. P., Timmermans, D. R. M., Knol, D. L., Bijnen, A. B., & Van Der Wal, G. (2007). Waiting for elective surgery: Effect on physical problems and postoperative recovery. *ANZ Journal of Surgery*, *77*(10), 892–898. https://doi.org/10.1111/j.1445-2197.2007.04268.x

Robling, M. R., Pill, R. M., Hood, K., & Butler, C. C. (2009). Time To talk? patient experiences of waiting for clinical management of knee injuries. *Quality and Safety in Health Care*, *18*(2), 141–146. https://doi.org/10.1136/qshc.2007.022475

Testi, A., Tanfani, E., Valente, R., Ansaldo, G. L., & Torre, G. C. (2008). Prioritizing surgical waiting lists. *Journal of Evaluation in Clinical Practice*, *14*(1), 59–64. https://doi.org/10.1111/j.1365-2753.2007.00794.x

van der Horst, A. Y., Trompetter, H. R., Pakvis, D. F. M., Kelders, S. M., Schreurs, K. M. G., & Bohlmeijer, E. T. (2019). Between hope and fear: A qualitative study on perioperative experiences and coping of patients after lumbar fusion surgery. *International Journal of Orthopaedic and Trauma Nursing*, *35*(July), 100707. https://doi.org/10.1016/j.ijotn.2019.07.003

Walker, S., Hopman, W. M., Carley, M. E., Mann, E. G., & Van Den Kerkhof, E. G. (2016). Healthcare use for pain in women waiting for gynaecological surgery. *Pain Research and Management*, *2016*. https://doi.org/10.1155/2016/1343568

Wallis, J. A., & Taylor, N. F. (2011). Pre-operative interventions (non-surgical and non-pharmacological) for patients with hip or knee osteoarthritis awaiting joint replacement surgery - a systematic review and meta-analysis. *Osteoarthritis and Cartilage*, *19*(12), 1381–1395. https://doi.org/10.1016/j.joca.2011.09.001

Supplementary File S2. Papers included in review.

Publication	Country	Objective	Sample Size and Characteristics	Time waiting for Surgery	Methods	Key Findings
Johnson et al., 2014	UK	To explore how people with osteoarthritis experience time while waiting for surgery.	n= 24 people waiting for hip replacement.	Not reported.	Longitudinal qualitative interviews at 10 days pre and up to four weeks post operatively.	2 themes: one was unavoidable changes to use and passage of time. n=1 participant unable to do hobbies and others in employment (paid/unpaid) had to reduce or terminate employment. Time controlled by healthcare system.
Carr et al., 2014	Canada	To explore the experiences of people with longer (orthopaedic) and shorter (cardiac)surgery waiting time.	n= 22 patients waiting for hip, knee, shoulder replacement, or n= 10 waiting for cardiac surgery.	Orthopaedic patients waited 90 days for surgery. Cardiac waiting times not stated.	Longitudinal qualitative interviews; first during the first week of being on waiting list, second at the pre-op assessment on the day before surgery.	3 themes: one was role restrictions and impact on family. n=1 couldn't work (orthopaedic). Lack of agency/control over wait time made the anxiety worse. 3 important factors; physical discomfort, personal control, whether the waiting allows for positive and productive experiences.
Carr et al., 2017	Canada	To understand patients' experiences of the effects of waiting for surgery.	n= 22 patients waiting for hip, knee, shoulder replacement (of whom 6 were working), or n= 10 waiting for cardiac surgery.	Orthopaedic patients waited 90 days for surgery. Cardiac waiting times < 2 weeks.	Longitudinal qualitative interviews; first during the first week of being on waiting list, second at the pre-op assessment on the day before surgery. Secondary data analysis of data collected in Carr (2014).	Themes; "restriction" included role limitation e.g., work and career pathway plans. Only one example given. People with certainty about wait time experienced less distress while waiting. Social support important but unhelpful if people keep asking for "any news". Being resigned to waiting (fatalistic) resulted in greater well-being. Waiting could be a positive opportunity for some, not for those working.
Morris et al., 2018	Australia	To describe quality of life (QoL) outcome measures in patients waiting for outpatient orthopaedic care and how waiting impacts on QoL.	18 papers reporting on the impact of QoL of people waiting for hip or knee replacement surgery. n= 2486 patients.	Durations in days; 60 - 286. 14 papers reported waiting times > 18 weeks.	Systematic scoping review. Search conducted August 2017.	First scoping review to report on QoL while waiting for orthopaedic assessment. Evidence 10 years old, focus on hip/knee, limited QoL outcome measures, inconsistent findings; deterioration in QoL observed in 11/18 papers, improvement observed in sub-groups in 3/18. Social isolation increases while waiting. Waiting time in 17/18 papers defined as starting from when patient added to surgical waiting list. Only I/18 papers defined waiting from point of referral by GP.

Tsang et al., 2016	Canada	To explore the patient experience while waiting for endoscopic sinus surgery (ESS).	n= 26 people waiting for ESS	Mean waiting time 216 days (range 31-425).	Mixed methods: questionnaires completed by n= 26 and qualitative interviews with n= 15.	Work Productivity and Activity Impairment - General Health (WPAI-GH) tool used to assess the impact of condition on work and non-work activity.4.8% (mean) had time off work, 34.4% impaired at work. Non work activity impaired by mean of 50.8%. Qualitative interviews: 7 people identified impacts on social situation (too tired to socialise), 2 identified poor sleep as a big factor impacting work function.
de Gorter, 2020	UK	To discuss how Patient Reported Outcome Measures (PROMS) can objectively support prioritisation of elective surgery	People waiting for surgery during Covid-19	not applicable	Commentary Article	PROMS use condition specific and generic (EQ-5D) assessment tools. The EQ-5D does not assess impacts on sleep. Health Function would be a rational, data drive, ethical basis for prioritisation. Use the following KPIs; Baseline Score, Follow Up Score, % reporting health loss, % reporting maximum follow up score, Average Health Gain.
Derrett et al., 1999	New Zealand	To describe the experiences of people waiting for elective surgery	n= 47 people waiting for hip/knee replacement and n= 102 waiting for prostatectomy	n= 30/47 people waiting for hip/ knee replacement waited > 6/12 and n= 71/102 people waiting for prostatectomy waited > 6/12	Quantitative; questionnaires (SF-36 and condition specific).	Half waiting for joint replacement surgery had problems with work and leisure activities. No relationship between length of wait and symptom severity for prostate or joint replacement surgery. SF-36 does not ask about sleep problems, standard of living, sexual function, education, embarrassment from health problem, or religion which in turn will affect work/social function.
Oudhoff et al., 2007a	Netherlands	To investigate the judgments of patients, stakeholders, laypersons, and physicians on the priority of people on surgical waiting lists.	Former patients; n= 82 with varicose veins, n= 86 inguinal hernia, n= 89 gallstones. Also, n= 101 surgeons, n= 95 occupational physicians, and n= 65 general practitioners.	Not applicable	Participants given vignettes of people waiting for surgery for varicose veins, inguinal hernia, and gallstones	Physical symptoms and impact of work had greatest impact on priority, but the sum of non-physical factors was higher. Physical, social, psychological, and work-related impacts were assessed using custom designed tool.

Oudhoff et al., 2007b	Netherlands	To assess the impact of waiting for elective general surgery on the QoL and psychosocial health of patients.	Patients waiting for surgery: n= 176 varicose veins, n= 201 inguinal hernia, n= 128 gallstones in 27 hospitals.	Median waiting times (days): gallstones 111, inguinal hernia 115, and varicose veins 170.	Quantitative: cross sectional questionnaire. 5 domains explored including Impact on Social Functioning. EQ-5D to assess QoL.	Social activities affected in 39-48% and work problems experienced by 18-23%. 12% with gall stones needed sick leave and 9.8% had problems caring for dependents. Gall stones more problematic than hernia. VVs now not done on NHS. 20% with hernia needed adjustments to workplace. 30% with gallstones reported problems going out. 39% gallstones problems with leisure activities. 48% with hernia had problem with hobbies. Most negative attitude to waiting amongst those with gallstones.
Søreide et al., 2020	Norway	To understand the immediate and long-term impact of the Covid-19 pandemic on delivery of surgical services.	Search methods and number of publications included not presented.	not applicable	Scoping literature review of the impact of Covid-19 on the delivery of surgical services.	No data identified on the effects of surgical cancellation on patients, including wellbeing, physical, and emotional health, functional capacity. Also not known are the patient's potential economic consequences e.g., loss of work, sick leave, ability to maintain housing/tenancy.
Ackerman et al., 2005	Australia	To determine health related QoL, psychological distress, physical function and self-efficacy in people waiting for hip/knee replacements.	n= 214 people waiting for hip/knee surgery. 59% women, 41% men.	n= 85/214 on waiting list for mean of 176 days (range 82-276) not stated for other participants.	Quantitative: AQoL, Kessler Distress scale and WOMAC (orthopaedic) assessment tools	AQoL used to assess. 1/3 had stopped work due to arthritis - 81% of this group were aged < 65 years. 15% felt they were in a state worse than death. Women and those with low socioeconomic status had worse AQoL
Hilkhuysen et al., 2005	Netherlands	To assess the consequences of waiting for surgery and design a general conceptual model.	10 surgeons, four occupational physicians, three general practitioners, one internist and two representatives of patients' associations	not applicable	Qualitative interviews	Social consequences of waiting reported: treatment delay can affect normal social functioning in both leisure activities and employment. This can lead to altered relationships with family, friends, work colleagues. Waiting can lead to prolonged sick leave or adjustments to work. A strong social network can help people.
Palmer et al., 2005	UK	To investigate the occupational factors, including size of organisation, associated with job loss in patients awaiting surgery to the hip or knee joint.	n=370 people of working age on the waiting list at a district general hospital.	Median waiting times 4-9 months.	Quantitative questionnaire. Focus on disability and effects on employment. Physical disability questions derived from the SF-36 used	n= 82/278 in work (30%) had to leave their job due to symptoms while waiting. More people left if younger, waiting longer, non-sedentary work. The likelihood of job modifications was higher if there was an occupational health service. Non sedentary job, company < 10 staff, part-timer hours, longer duration of symptoms, age < 34 years more likely to have left their job due to symptoms.

Herrod et al., 2019	UK	To quantify the economic and psychosocial impact of the cancellation of operations due to winter pressures on their patients, their families, and the economy.	n= 339 people waiting for surgery in 5 hospitals (68% employed) between 1st November 2017 and 31st March 2018 when elective services were cancelled due to winter pressures.	Not stated.	Quantitative questionnaire plus free text analysed thematically. WES-PI survey. First study in England to look at patients' economic and psychological viewpoints.	n= 163/399 of working age (<65 years) and of these n= 111 (68%) were employed n= 69/111 (62%) of those working could return to work during the time scheduled off for surgery. Total loss of workdays was 329. Mean loss of 5 working days (+/-10). Additional working days lost by 54% (+/-10). 33% of 111 family needed time off work (mean 5, +/- 7) totalling 581 days.
Ackerman et al., 2011	Australia	To investigate changes in health related QoL, health status and psychological distress among people waiting hip/ knee replacements.	n= 134 waiting for joint replacement surgery	69% waited longer than 6 months for surgery (median 286 days, range 169-375).	Quantitative prospective cohort: AQoL, Kessler Distress scale and WOMAC (orthopaedic) assessment tools	AQoL used to assess. Includes level of assistance needed for personal care and household tasks, relationships with other, social isolation, ability to perform role in family and sleep. Overall scores showed a deterioration in QoL from baseline to preadmission in 53%. Pain (84%), fatigue (76%), QoL (73%), confidence in managing health (55%) worsened during the wait. Overall deterioration in HRQoL without a corresponding change in health status or psychological distress indicates that the waiting time per se was problematic.
Brownlow et al., 2001	UK	To assess aspects of the physical and social function and mental well-being of patients waiting for hip replacements.	n= 95 people waiting for hip replacement surgery	Median time waiting 6 months range (1-26).	Cross sectional study. Questionnaires using existing tools SF 36, Postel (hip score), GHQ and HADs (mental health).	No evidence that mental health or social function were worst in those who waited longest. Possibly because: people are only listed for surgery when symptoms are already severe; if the disorder is chronic then physical deterioration may be slow; those with more distress asked repeatedly to have their surgery brought forward; some people have better pain management.
Carr et al., 2009	Canada	To perform a systematic review of the impact of waiting for surgery from the patient perspective, with a focus on maximum tolerance, quality of life, and the nature of the waiting experience.	n= 27 papers included. Research conducted from patients' standpoints.	Not stated.	Systematic literature review including both quantitative and qualitative papers.	n= 11 studies about waiting times (including cardiac): pain, loss of mobility, time to prepare. n=7 studies about QoL: pain, mobility, loss of finances, being alone, leisure activity, loss of dignity. Longer waits associated with a decreased likelihood of returning to work. Support (practical and emotional) from family and friends was important. n= 9 studies about patients experiences while waiting for surgery: younger and blue-collar workers had more economic hardship with delays (cardiac surgery).

Conner-Spady et al., 2007	Canada	To obtain patients' perspectives on acceptable waiting times for hip or knee replacement surgery.	n= 303 people waiting for hip or knee surgery	Median waiting time was 17 weeks.	Questionnaire with closed and open questions.	Median acceptable waiting time was 13 weeks, based on pain and loss of mobility. Increase in pain and decrease in ADLs important consequences - loss of work and poor sleep due to pain and loss of mobility was reported qualitatively, also reliance on strong prescribed drugs.
Desmeules et al., 2009	Canada	To measure pain, stiffness, function, and HRQoL in patients at enrolment on waiting list for knee replacement.	n= 197 people from 3 hospitals. Cross section.	Not stated. Participants enrolled when first placed on waiting list.	Quantitative: questionnaires to measure function (WOMAC) and the SF-36 for QoL.	Married subjects had better QoL regarding physical role limitations (i.e., people not living alone). Use of a walking aid and high BMI - worse QoL. Social functioning score of participants vs general population was 40.9 vs 87. Low social support also associated with low mental QoL. Worse QoL with bilateral knee pain. Suggests pre-habilitation programmes to reduce psychological distress and weight loss management. Problems with physical and non-physical symptoms begin prior to being listed for surgery.
Oudhoff et al., 2004	Netherlands	To assess current literature about the effects of delayed surgery for varicose veins, inguinal hernia, gall stones and breast cancer in terms of physical, psychological, and social aspects.	n=23 papers identified via Medline and Embase between January 1985 and September 2003.	Not applicable.	Descriptive literature review.	Social and psychological impacts of varicose veins or hernia are not a high priority, but cholecystitis (with more pain) does. Also, improved social functioning in women waiting for breast biopsy rather than cholecystectomy. Authors call for clear priority criteria and a specific assessment of the health impact while waiting.
Sjöling et al., 2005	Sweden	To explore peoples' lived experience of being on the waiting list for arthroplastic surgery of the knee or hip and its impact on daily life.	n= 18 people (9 men & 9 women) aged 51-82 years.	Median waiting time for surgery 71 weeks (range 30-65).	Qualitative interviews.	Disability, loss of dignity, feeling neglected, unable to walk anywhere, adapt ADLs, stigma, excluded from society. Life on hold. Meaningless life, unable to do hobbies, things that give life meaning restricted by pain. Not knowing how long the wait would be was difficult. No mention of work (Paid or family roles) or economic impacts. People who could focus on their abilities rather than deficiencies found it easier to cope.

Ackerman, I. N., Bennell, K. L., & Osborne, R. H. (2011). Decline in Health-Related Quality of Life reported by more than half of those waiting for joint replacement surgery: A prospective cohort study. *BMC Musculoskeletal Disorders*, *12*. https://doi.org/10.1186/1471-2474-12-108

Ackerman, I. N., Graves, S. E., Wicks, I. P., Bennell, K. L., & Osborne, R. H. (2005). Severely compromised quality of life in women and those of lower socioeconomic status waiting for joint replacement surgery. *Arthritis Care and Research*, *53*(5), 653–658. https://doi.org/10.1002/art.21439

Brownlow, H. C., Benjamin, S., Andrew, J. G., & Kay, P. (2001). Disability and mental health of patients waiting for total hip replacement. *Annals of the Royal College of Surgeons of England*, *83*(2), 128–133.

Carr, T., Teucher, U. C., & Casson, A. G. (2014). Time while waiting: Patients' experiences of scheduled surgery. *Qualitative Health Research*, *24*(12), 1673–1685. https://doi.org/10.1177/1049732314549022

Carr, T., Teucher, U., & Casson, A. G. (2017). Waiting for scheduled surgery: A complex patient experience. *Journal of Health Psychology*, *22*(3), 290–301. https://doi.org/10.1177/1359105315603464

Carr, T., Teucher, U., Mann, J., & Casson, A. G. (2009). *PRBM_7652_waiting-for-surgery-from-the-patient-perspective*. 107–119.

Conner-Spady, B. L., Johnston, G. H., Sanmartin, C., McGurran, J. J., Noseworthy, T. W., Weiler, R., Brown, J., Bryden, C., Calder, D., Donnelly, L., Gander, L., Johnson, D., Larsen, D., McRae, S., Ogrady, M., & Truscott, T. (2007). A bird can't fly on one wing: Patient views on waiting for hip and knee replacement surgery. *Health Expectations*, *10*(2), 108–116. https://doi.org/10.1111/j.1369-7625.2006.00425.x

de Gorter, J. (2020). *How patient reported outcome measures (PROMS) can help prioritisation of elective surgery and value based healthcare post Covid-19*. Linkedin. https://www.linkedin.com/pulse/how-patient-reported-outcome-measures-proms-can-help-value-de-gorter/

Derrett, S., Paul, C., & Morris, J. M. (1999). Waiting for elective surgery: Effects on health-related quality of life. *International Journal for Quality in Health Care*, *11*(1), 47–57. https://doi.org/10.1093/intqhc/11.1.47

Desmeules, F., Dionne, C. E., Belzile, É., Bourbonnais, R., & Frémont, P. (2009). Waiting for total knee replacement surgery: Factors associated with pain, stiffness, function and quality of life. *BMC Musculoskeletal Disorders*, *10*(1), 1–10. https://doi.org/10.1186/1471-2474-10-52

Herrod, P. J. J., Adiamah, A., Boyd-Carson, H., Daliya, P., El-Sharkawy, A. M., Sarmah, P. B., Hossain, T., Couch, J., Sian, T. S., Wragg, A., Andrew, D. R., Parsons, S. L., Lobo, D. N., Johnson, B., Koh, A., Kushairi, A., Lewis-Lloyd, C., Roslan, F., Thompson, A., … Singh, B. (2019). Winter cancellations of elective surgical procedures in the UK: A questionnaire survey of patients on the economic and psychological impact. *BMJ Open*, *9*(9), 1–8. https://doi.org/10.1136/bmjopen-2018-028753

Hilkhuysen, G. L. M., Oudhoff, J. P., Rietberg, M., van der Wal, G., & Timmermans, D. R. M. (2005). Waiting for elective surgery: A qualitative analysis and conceptual framework of the consequences of delay. *Public Health*, *119*(4), 290–293. https://doi.org/10.1016/j.puhe.2004.05.020

Johnson, E. C., Horwood, J., & Gooberman-Hill, R. (2014). Conceptualising time before surgery: The experience of patients waiting for hip replacement. *Social Science and Medicine*, *116*, 126–133. https://doi.org/10.1016/j.socscimed.2014.06.037

Mats Sjöling, R. N., Ylva Ågren, R. N., Olofsson, N., Ove Hellzén, R. N., & Kenneth Asplund, R. N. (2005). Waiting for surgery; living a life on hold - A continuous struggle against a faceless system. *International Journal of Nursing Studies*, *42*(5), 539–547. https://doi.org/10.1016/j.ijnurstu.2004.09.009

Morris, J., Twizeyemariya, A., & Grimmer, K. (2018). What is the current evidence of the impact on quality of life whilst waiting for management/treatment of orthopaedic/musculoskeletal complaints? A systematic scoping review. *Quality of Life Research*, *27*(9), 2227–2242. https://doi.org/10.1007/s11136-018-1846-z

Oudhoff, J. P., Timmermans, D. R. M., Knol, D. L., Bijnen, A. B., & Van Der Wal, G. (2007). Waiting for elective general surgery: Impact on health related quality of life and psychosocial consequences. *BMC Public Health*, *7*, 1–10. https://doi.org/10.1186/1471-2458-7-164

Oudhoff, Jurriaan P., Timmermans, D. R. M., Bijnen, A. B., & Van Der Wal, G. (2004). Waiting for elective general surgery: Physical, psychological and social consequences. *ANZ Journal of Surgery*, *74*(5), 361–367. https://doi.org/10.1111/j.1445-1433.2004.02998.x

Oudhoff, Jurriaan P., Timmermans, D. R. M., Knol, D. L., Bijnen, A. B., & Van der Wal, G. (2007). Prioritising patients on surgical waiting lists: A conjoint analysis study on the priority judgements of patients, surgeons, occupational physicians, and general practitioners. *Social Science and Medicine*, *64*(9), 1863–1875. https://doi.org/10.1016/j.socscimed.2007.01.002

Palmer, K. T., Milne, P., Poole, J., Cooper, C., & Coggon, D. (2005). Employment characteristics and job loss in patients awaiting surgery on the hip or knee. *Occupational and Environmental Medicine*, *62*(1), 54–57. https://doi.org/10.1136/oem.2004.014977

Søreide, K., Hallet, J., Matthews, J. B., Schnitzbauer, A. A., Line, P. D., Lai, P. B. S., Otero, J., Callegaro, D., Warner, S. G., Baxter, N. N., Teh, C. S. C., Ng-Kamstra, J., Meara, J. G., Hagander, L., & Lorenzon, L. (2020). Immediate and long-term impact of the COVID-19 pandemic on delivery of surgical services. *British Journal of Surgery*, *107*(10), 1250–1261. https://doi.org/10.1002/bjs.11670

Tsang, G. F. Z., McKnight, C. L., Kim, L. M., & Lee, J. M. (2016). Exploring the psychological morbidity of waiting for sinus surgery using a mixed methods approach. *Journal of Otolaryngology - Head and Neck Surgery*, *45*(1), 1–7. https://doi.org/10.1186/s40463-016-0149-z
